# Navigating Portal Hypertension and Splenomegaly: A Clinical Encounter With an Unusual Variant of Cruveilhier-Baumgarten Syndrome

**DOI:** 10.7759/cureus.62431

**Published:** 2024-06-15

**Authors:** Abhishek Sharma, Pulkit Mehrotra, Sivaprakash Varadan, R.B Sudagar Singh

**Affiliations:** 1 General Medicine, Sri Ramachandra Institute of Higher Education and Research, Chennai, IND; 2 Internal Medicine, Sri Ramachandra Institute of Higher Education and Research, Chennai, IND

**Keywords:** chronic liver disease (cld), cruveilhier-baumgarten syndrome, splenomegaly, gastro-esophageal varices, portal hyperetnsion

## Abstract

Cruveilhier-Baumgarten syndrome presents a rare manifestation of portal hypertension characterized by a portosystemic shunt through a dilated paraumbilical vein, typically accompanied by classical signs such as caput medusae and a venous hum. We report a compelling case of a 41-year-old male presenting with portal hypertension, exhibiting clinical and radiological features of Cruveilhier-Baumgarten syndrome but notably lacking the characteristic venous hum. Clinical examination revealed moderate splenomegaly with prominent dilated veins and venous thrill but no caput medusae. Laboratory investigations indicated thrombocytopenia and esophageal varices on upper GI endoscopy. Imaging studies confirmed portal hypertension with findings consistent with Cruveilhier-Baumgarten syndrome, including a dilated paraumbilical vein and splenic artery aneurysms, along with the unexpected absence of a venous hum. Despite the classical radiological features, our patient did not present with hematemesis, possibly attributed to the presence of paraumbilical veins. This case highlights the diagnostic challenges and atypical presentations of Cruveilhier-Baumgarten syndrome, emphasizing the importance of comprehensive clinical evaluation and imaging modalities in its diagnosis and management. Management strategies primarily focus on addressing portal hypertension and underlying liver disease. This case underscores the need for further research to elucidate the varied clinical presentations and pathophysiology of Cruveilhier-Baumgarten syndrome variants, enhancing our understanding and management of this rare entity.

## Introduction

Cruveilhier-Baumgarten syndrome is characterized by a portosystemic shunt from the left portal vein via a dilated paraumbilical vein that terminates at the umbilicus. A caput medusae and a venous hum or abdominal bruit are classical signs of this syndrome, but in our case, the absence of the hum makes it an unusual presentation [1]. We present an interesting case of a 41-year-old male with portal hypertension with clinical and radiological features of Cruveilhier-Baumgarten syndrome with an absent venous hum.

## Case presentation

A 41-year-old male, thin-built, presented with a history of generalized weakness, abdominal pain, and dark brown-colored stool for three days. He had no known comorbidities. He was non-alcoholic and a non-smoker. On examination, the patient was conscious and oriented. He was afebrile. There was no pedal edema, jaundice, or clubbing. His pulse was 96 bpm, his respiratory rate was 20 per minute, and his sitting blood pressure was 110/70 mmHg. Per abdomen, examination revealed moderate splenomegaly with prominent dilated veins and a venous thrill with no venous hum. There were no dilated superficial vessels around the umbilicus. There were no systemic abnormalities with the cardiac, respiratory, or nervous systems.

His laboratory investigations revealed a hemoglobin of 11.1gm/dl, a leukocyte count of 2,200/cmm, and low platelets (35,000 cells/cmm). No hemoparasites were seen in the peripheral smear. Random blood sugar was 108 mg/dl. His liver function was within normal limits (total bilirubin: 0.83 mg/dl, SGOT: 53 U/L, SGPT: 45 U/L, total proteins: 7.3 gm/dl, serum albumin: 3.5 gm/dl, and serum globulin: 3.8 gm/dl). The alkaline phosphatase level was 126 U/L. His renal function was normal. The urine routine was unremarkable. Viral markers for HIV, HCV, and HBV were negative. Fever panel testing done for dengue, malaria, scrub typhus, and leptospirosis was negative. In evaluation with two-dimensional cardiac echocardiography, there was trivial mitral, tricuspid, and pulmonary regurgitation with normal ejection fraction and normal pulmonary artery pressure. Abdominal ultrasound revealed a dilated portal and splenic veins, moderate splenomegaly, and few peri-splenic collaterals. The liver was small in size and had coarse echotexture, with no abnormalities in other organs. Fibroscan showed a KpA value of 21.6, suggestive of F4 fibrosis. Upper GI endoscopy showed high-grade esophageal varices with red color signs and severe portal hypertensive gastropathy. Prophylactic endoscopic variceal ligation was done in view of the increased risk of upper GI bleeding.

**Figure 1:**
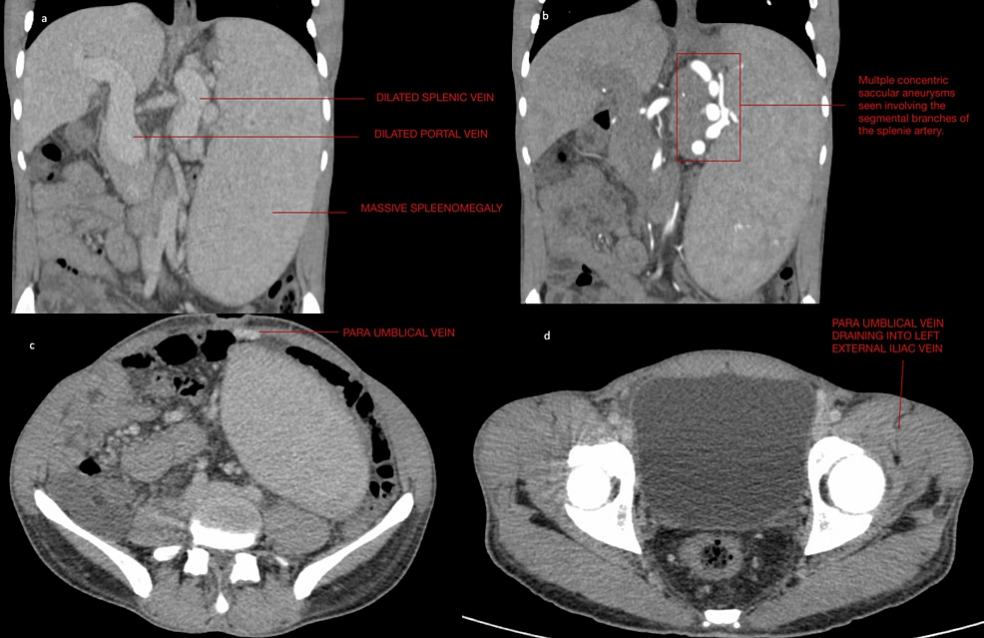
CECT scan showing (a) enlarged portal and splenic veins along with massive splenomegaly; (b) multiple aneurysms at the splenic artery; (c) dilated paraumbilical vein; and (d) paraumbilical vein draining into the left external iliac vein CECT, contrast-enhanced CT

Based on clinical findings and investigations, a provisional diagnosis of cirrhosis of the liver with portal hypertension was made. Further evaluation with a contrast-enhanced CT (CECT) scan showed a smaller liver (8.8 cm) with no focal parenchymal lesions. The main portal vein and its branches were dilated. There was focal ectasia of the main portal vein at the level of portal confluence, which measured 28 mm, indicating portal vein aneurysm or portal varix. A dilated and tortuous recanalized paraumbilical vein along the course of the anterior wall draining into the left external iliac vein was seen. These recanalized paraumbilical veins were also seen up to the umbilicus. The spleen was grossly enlarged (22 cm), and the splenic index was 3,742. The splenic vein (20 mm) and splenic artery (10 mm) were dilated and tortuous, along with multiple concentric aneurysms of varying sizes on segmental branches of the splenic artery (Figure 1). Multiple venous collateral formations were also noted around the umbilicus. There were minimal ascites, with no abnormalities in other organs. In light of clinical details and imaging findings in our case, the diagnosis of cirrhosis of the liver with portal hypertension with all the radiological features and clinical signs of Cruveilhier-Baumgarten syndrome except venous hum suggests a variant of the syndrome. Supportive management was done with IV fluids, IV antibiotics, and nonselective beta-blockers.

## Discussion

Portal hypertension leads to the opening of collaterals to carry portal blood into the systemic veins. The paraumbilical vein is one of the important collateral pathways. The umbilical vein in infancy becomes the ligamentum teres after birth [2]. Small paraumbilical vessels run adjacent to the ligamentum teres, which may represent the vasa vasorum that connects the veins around the umbilicus to the portal vein. The paraumbilical flow generally returns to the systemic circulation, mostly via the inferior epigastric veins [3]. In 1833, Pegot reported a case of portal hypertension in which a loud venous hum was heard at the umbilicus for the first time. It was elaborated by Cruveilhier, who found on autopsy that the venous murmur was due to collateral circulation through a widely patent umbilical vein. In 1908, Baumgarten reported a similar case. The name Cruveilhier-Baumgarten syndrome was given by Armstrong, who reviewed these cases [1].

Cruveilhier-Baugarten syndrome refers to the recanalization and dilatation of the paraumbilical vein with prominent periumbilical portosystemic collaterals in patients with cirrhosis and portal hypertension. A characteristic finding at clinical examination is the presence of caput medusae (tortuous abdominal wall varices) and of a loud venous murmur (the Cruveilhier-Baumgarten bruit) that can be heard over the upper abdomen with a palpable thrill [4,5]. Surprisingly, despite having a large, dilated, tortuous paraumbilical vein, our patient neither had the visible caput medusae nor the venous hum. Cruveilhier-Baumgarten disease, on the other hand, refers to congenital failure of the closure of umbilical veins, causing distension of paraumbilical veins, with little or no evidence of liver disease found on imaging and liver biopsy [6].

Multidetector CT (MDCT) is the imaging modality of choice to delineate this portosystemic collateral pathway with great precision. On MDCT, Cruveilhier-Baumgarten syndrome is diagnosed by the presence of a dilated tortuous paraumbilical vein, which arises from the left portal vein branch, traverses the falciform ligament, and forms a network of dilated periumbilical veins. The blood eventually drains into the systemic circulation via the superficial and deep epigastric veins to the external iliac or femoral vein [5,7]. Since portosystemic varicose veins develop by distention and elongation of the preexisting small veins, the walls of variceal veins are thin. These vessels break easily and are difficult to repair. Inadvertent injury to the vessels during paracentesis or abdominal surgeries can cause massive, life-threatening bleeding [3,8]. Spontaneous hemorrhage can also occur in these vessels. The recanalized vessels may prevent bleeding from esophageal varices [1,8]. It is a fact worth noting that our patient did not have episodes of hematemesis despite having large esophageal varices and portal gastropathy, which may be attributed to the presence of paraumbilical veins.

## Conclusions

Our patient’s case is interesting because, despite having Cruveilhier-Baumgarten syndrome, i.e., a large tortuous paraumbilical vein draining into an external iliac vein, the Cruveilhier-Baumgarten sign, i.e., a venous murmur or hum, was absent on auscultation. This absence can be attributed to lower portal pressure, which may also explain the absence of an episode of hematemesis despite high-grade varices. Also, CECT showed uncommon findings of multiple small splenic artery aneurysms as well as portal vein aneurysms, which may also have contributed to the absence of venous hum despite the classical feature of Cruveilhier-Baumgarten syndrome. Patients with Cruveilhier-Baumgarten syndrome are managed on the basis of portal hypertension and underlying primary disease. Similarly, our case was managed with supportive treatment.
